# Body mass index and weight loss in patients submitted to orthognathic surgery: a prospective study

**DOI:** 10.1590/2177-6709.28.5.e2323107.oar

**Published:** 2023-11-10

**Authors:** Leonardo BENATO, Alice Vanzela MIOTTO, Romulo Lazzari MOLINARI, Bernardo OLSSON, Lígia de Oliveira CARLOS, Rubia Daniela THIEME, Maria Eliana Madalozzo SCHIEFECKER, Nelson Luis Barbosa REBELLATO, Rafaela SCARIOT, Leandro Eduardo KLÜPPEL

**Affiliations:** 1 Federal University of Paraná, School of Dentistry, Department of Oral and Maxillofacial Surgery (Curitiba/PR, Brazil).; 2 Federal University of Paraná, Clinical Surgery Department (Curitiba/PR, Brazil).; 3 Federal University of Paraná, Public Policy, Department of Nutrition (Curitiba/PR, Brazil).; 4 Federal University of Paraná, Department of Nutrition (Curitiba/PR, Brazil).

**Keywords:** Body mass index, Weight loss, Dentofacial deformities, Orthognathic surgery

## Abstract

**Objective:**

To compare the body mass index (BMI) and the weight loss (WL) in patients with dentofacial deformities who underwent monomaxillary versus bimaxillary orthognathic surgery.

**Materials and Methods:**

This prospective longitudinal study included 69 patients with dentofacial deformities who underwent surgical orthodontic treatment. Patients were divided into two groups according to the type of orthognathic surgery: monomaxillary or bimaxillary. A preoperative nutritional assessment based on BMI was performed; the percentage of involuntary WL between the preoperative and postoperative periods was also calculated. Data were collected at preoperative and 10, 40, and 90 days postoperative (PO). Statistical analysis was performed using SPSS 17.0 (IBM Corp., Armonk, NY, USA), and data are reported with 95% confidence interval.

**Results:**

According to BMI, patients who underwent monomaxillary surgery presented: underweight = 2.6%, normal weight = 51.3%, overweight = 35.9%, and obese = 10.3%. The subjects who underwent bimaxillary surgery presented: normal weight = 43.3%, overweight = 36.7%, and obese = 20%. BMI was similar between the groups at all time points (preoperative, *p*= 0.237; 10 days PO, *p*= 0.325; 40 days PO, *p*= 0.430; and 90 days PO, *p*= 0.609). All patients lost weight postoperatively, and WL was similar among the PO measurements (*p*= 0.163).

**Conclusions:**

Although both monomaxillary and bimaxillary orthognathic surgeries resulted in WL and lower BMI, there was no statistically significant difference in these metrics between the two types of surgery.

## RESUMO

**Objetivo:**

Comparar o Índice de Massa Corporal (IMC) e a perda de peso (PP) de pacientes com deformidades dentofaciais após cirurgia ortognática monomaxilar e bimaxilar.

**Material e Métodos:**

Foi realizado um estudo longitudinal prospectivo em 69 pacientes com deformidade dentofacial submetidos a tratamento cirúrgico-ortodôntico. Os pacientes foram divididos em dois grupos: cirurgia ortognática monomaxilar e bimaxilar. Foi realizada avaliação nutricional pré-operatória de acordo com o IMC. Foi calculada a porcentagem de PP involuntária entre o pré e o pós-operatório (PO). A coleta de dados foi realizada no pré-operatório e aos 10, 40 e 90 dias PO. A análise estatística foi realizada no *software* SPSS v. 17.0, com intervalo de confiança de 95%.

**Resultados:**

De acordo com o IMC, os indivíduos submetidos à cirurgia monomaxilar apresentaram: baixo peso = 2,6%, peso normal = 51,3%, sobrepeso = 35,9% e obesidade = 10,3%. Entre os indivíduos submetidos à cirurgia bimaxilar, 43,3% estavam com peso normal, 36,7% estavam com sobrepeso e 20% eram obesos. O IMC foi semelhante em todos os períodos (pré-operatório, *p*= 0,237; 10 dias PO, *p*= 0,325; 40 dias PO, *p*= 0,430; e 90 dias PO, *p*= 0,609). Todos os pacientes perderam peso no pós-operatório. A PP foi semelhante entre os tempos de PO (*p*= 0,163).

**Conclusões:**

A cirurgia ortognática monomaxilar ou bimaxilar pode causar redução do peso corporal e diminuição no IMC, mas não há diferença estatisticamente significativa entre esses dois tipos de cirurgia em relação à PP e ao IMC.

## INTRODUCTION

Dentofacial deformities (DDF) are defined by deviations from normal facial proportions and, when sufficiently serious, dental relations develop into disabilities.^1^ Orthognathic surgery performed in conjunction with orthodontic treatment is commonly performed to correct skeletal irregularities and realign the maxillomandibular relationship, to improve occlusal function and facial esthetics.^2^ ^,^ ^3^ Through this therapeutic approach, important dentofacial changes are achieved, which not only correct functional and aesthetic aspects, but also yield emotional benefits.^4^ It is common for patients to report improvement in their self-esteem, self-image, and social integration after undergoing this surgery.^4^ ^-^ ^6^ Orthognathic surgery can be used to treat DDF only in the maxilla or mandible, or combined in maxilla and mandible.^1^ ^,^ ^6^

During the postoperative period, patients cannot properly move their jaws to chew. Only in the following weeks after the surgery they can change from a liquid to a liquid-pasty diet, but they are still, nevertheless, unable to chew hard or crunchy foods for several weeks.^7^ In the postoperative period, the use of elastics placed between the jaws, designed to make small adjustments to guide occlusion, is common; however, elastics can also impair chewing.^8^ The result of these interventions may expose patients to the risk of becoming nutritionally deficient and dehydrated, with complaints of pain, discomfort, anorexia, and nausea, as well as significant weight loss (WL).^7^ ^-^ ^10^

WL is considered to be one of the side effects of orthognathic surgery due to immediate postoperative functional limitations and nutritional restriction.^11^ ^,^ ^12^ It has been suggested that changes in body mass index (BMI) resulting from metabolic changes should be diagnosed, to identify and avoid early health risks.^11^ In the literature, there is a lack of systematic documentation of the WL experienced by patients during the first few weeks or months of the time required for postoperative recovery after orthognathic surgery.^7^ Thus, although the postoperative period for orthognathic surgery is not ideal for nutrition,^12^ the impact on BMI and WL has not been extensively studied. Additionally, bimaxillary surgery is commonly associated with the worst postoperative period and poor nutrition.^13^

Therefore, knowledge regarding nutritional status and related factors in the early postoperative period following orthognathic surgery is important for perioperative management.^10^ Previous studies have demonstrated the importance of investigating WL and BMI after orthognathic surgery. However, more studies are needed to fill important information gaps in the literature.^7^ Therefore, the purpose of the present study was to compare BMI and WL in patients undergoing monomaxillary *versus* bimaxillary orthognathic surgery.

## MATERIAL AND METHODS

### ETHICS CONSIDERATIONS

This study was approved by the Ethics Committee of the Federal University of Paraná (CAAE:24855413.0.0000.0102) and was performed in accordance with the Declaration of Helsinki. All participants who agreed to participate in the study provided informed written consent.

### STUDY DESIGN AND PARTICIPANTS

The present investigation was designed as a prospective longitudinal clinical study, which included a convenience sample of patients. Participants were invited to participate in a public oral and maxillofacial reference center. The inclusion criteria were adult patients (≥ 18 years of age) of both sexes with DDF, who underwent monomaxillary or bimaxillary orthognathic surgery between 2013 and 2015. Syndromic patients, those who had previously undergone orthognathic surgery, and those who missed any of the evaluations were excluded from the study. 

The surgeries were performed by residents in their third year of service, and supervised by the chief surgeon. During the postoperative period, the patients remained in hospital for two days, and were maintained on a liquid diet in the first week, evolving to a soft diet from the second week thereafter. After 40 days, patients were instructed to return to their normal diet. Standard dietary postoperative recommendations were provided to the patients.

All patients used intermaxillary elastics to guide occlusion for two to six weeks, although patients were able to remove the elastics to eat.

### DATA COLLECTION

Data including sex, age, type of surgery, weight, and height were collected. Patients were divided into two groups according to the type of surgery: monomaxillary, involving only the maxilla or mandible; or bimaxillary, involving the maxilla and mandible. The Le Fort I technique and bilateral split sagittal osteotomy were performed on the maxilla and mandible, respectively. 

Patient weight was assessed using a previously calibrated commercially available portable digital scale (BC548 Ironman, Tanita, Arlington Heights, IL, USA) with 0.1 kg measurement intervals. Patient height was measured using a commercially available portable stadiometer (WCS, Cardiomed, Brazil) with a maximum capacity of 200 cm at 0.5 cm increments. These data were obtained at four time points: preoperative and at 10, 40, and 90 postoperative (PO) days. The methods for measuring current and actual height recommended by the Brazilian Ministry of Health were used. Data regarding patient weight and height were used to calculate BMI and WL. BMI was calculated using the following equation:

$$BMI\;\left(kg/m^{2}\right)\;=\;weight\;\left(kg\right)\;/\;height\;{\left(m\right)}^{2}$$

Patients were classified according to BMI, as follows: underweight (BMI < 18.5 kg/m^2^); normal weight (BMI = 18.5-24.9 kg/m^2^); overweight (BMI 25.0-29.9 kg/m^2^); and obese (BMI > 30.0 kg/m^2^)^16^ ^,^ ^17^. BMI values were calculated preoperatively and at 10, 40 and 90 days postoperatively. 

WL was calculated from current weight in the preoperative period and at each postoperative interval. WL is expressed as the percentage of involuntary WL, using the equation:

WL=(preoperativeweight[kg]–postoperativeweight[kg])×100/preoperativeweight.

Statistical analyses were performed using SPSS v. 17.0 (IBM Corporation, Armonk, NY, USA). Data analysis was performed according to characteristics of a normal distribution of variables (Shapiro-Wilk test) and expressed as mean ± standard deviation (SD). Bivariate analysis was performed using an independent sample *t*-test; differences with *p* < 0.05 were considered to be statistically significant. The chi-squared test was used to compare sex and type of malocclusion between the monomaxillary and bimaxillary orthognathic surgery groups.

## RESULTS

**Figure 1:**
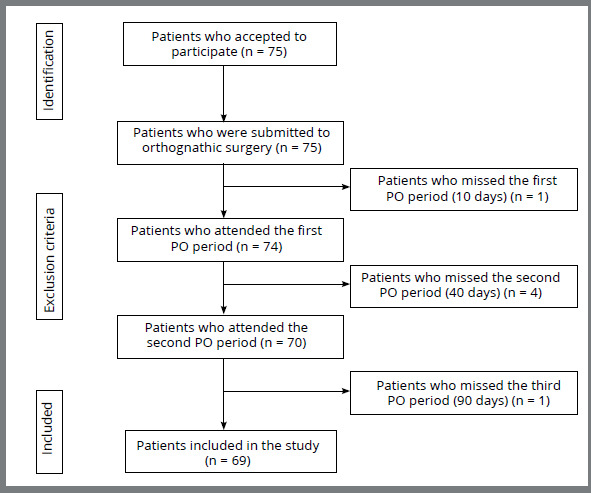
Flow-diagram illustrating patient inclusion and exclusion from the study.

Seventy-five patients were enrolled in the study; however, 6 were excluded because they did attend the scheduled follow-up visit(s) (Fig 1). As such, the final sample comprised 69 patients (39 female [56.5%], 30 male [43.5%]), with a mean (± SD) age of 30.7 ± 9.7 years. Regarding the type of surgery, 39 (56.5%) and 30 (43.5%) patients comprised the monomaxillary and bimaxillary groups, respectively. In the monomaxillary surgery group, 26 patients underwent maxillary advancement and 13 underwent mandibular advancement. The most prevalent DDF was skeletal Class III malocclusion (n = 48 [69.5%]).

### BMI

Data regarding sex, age, initial BMI, and malocclusion in patients who underwent monomaxillary and bimaxillary orthognathic surgery are summarized in Table 1. Although no statistically significant difference was found (p > 0.05), the sample mainly comprised females and skeletal Class III patients. 

**Table 1:** Distribution of sex, age, initial body mass index (BMI), and malocclusion between patients who underwent monomaxillary or bimaxillary orthognathic surgery.

	Monomaxillary n (%)	Bimaxillary n (%)	***p*-value**
Sex			0.983*
Male	17 (43.6)	13 (43.3)	
Female	22 (56.4)	17 (56.7)	
Age - mean (SD)	30.3 (9.5)	28.3 (7.6)	0.368^#^
Initial BMI - mean (SD)	25.4 (4.4)	26.3 (4.5)	0.237^#^
Malocclusion			0.834*
Angle Class II	17 (43.6)	22 (45.8)	
Angle Class III	22 (56.4)	26 (54.2)	

BMI = Body Mass Index. SD = Standard Deviation.

*Chi-square test; ^#^ independent samples t-test.

The variations in BMI in each group over time are summarized in Table 2. BMI was similar at all time points between the groups that underwent monomaxillary or bimaxillary surgery.

**Table 2:** Body mass index (BMI) for the different surgical procedures over time.

	Monomaxillary Mean (SD)	Bimaxillary Mean (SD)	*p* -value*
Preoperative	25.4 (4.37)	26.3 (4.52)	0.237
10 days PO	24.4 (4.27)	25.0 (4.41)	0.325
40 days PO	24.2 (3.96)	25.0 (4.37)	0.430
90 days PO	24.8 (4.07)	25.2 (4.19)	0.609

BMI = Body Mass Index; PO = post-operative; SD = standard deviation; *independent samples *t*-test.

### WL

Data regarding variation in WL in each group over time are summarized in Table 3. It is noteworthy that all patients, regardless of surgical group allocation (i.e., monomaxillary *versus* bimaxillary), lost weight at all time points evaluated, with similar WL values. 

**Table 3:** Weight loss among patients undergoing the different surgical procedures over time.

	Monomaxillary Mean (SD)	Bimaxillary Mean (SD)	*p* -value*
Preoperative	0 (-)	0 (-)	-
PO 10 days	3.9 (1.71)	5.0 (1.72)	0.074
PO 40 days	5.3 (2.45)	5.9 (3.41)	0.531
PO 90 days	3.9 (2.78)	5.4 (2.65)	0.131

PO = post-operative; SD = standard deviation; * independent samples *t*-test.

## DISCUSSION

A vast body of literature has addressed the issue of orthognathic surgery using new application techniques and materials. However, studies on nutritional assessment in patients undergoing this procedure have been restricted to a small range of scientific articles, with little information available. Most articles report, in addition to nutritional assessment, nutritional interventions,^10^ ^,^ ^12^ which differs from studies that focus on preoperative nutritional diagnosis, WL, and changes in BMI after surgery.^10^ ^,^ ^13^ Acknowledging that patients undergoing orthognathic surgery experience drastic changes in weight in a short time, the present study was designed in collaboration with the nutrition department of the university to provide a more accurate diagnosis of the nutritional status of these patients.

All patients participating in the present study had DDF, with orthognathic surgical treatment indicated for resolution. Sixty-nine patients were evaluated, being 39 (56.5%) female. The proportion of females in this study is consistent with previous studies,^1^ ^,^ ^13^ ^,^ ^14^ confirming that more females seek treatments that alter facial esthetics. The higher proportion of females also explains the higher prevalence of skeletal Class III patients; since Class III facial profiles are considered less esthetic favorably among females than for males.^1^

Regarding preoperative nutritional evaluation, it was considered the possibility that DDF could be associated with some degree of malnutrition, because difficulty with chewing experienced by these patients could limit the quantity and/or quality of dietary intake. However, it was observed that only 1 patient was underweight, which makes it clear that the presence of a deformity does not necessarily interfere with nutritional status. Furthermore, patient BMI and preoperative weight should be compared with DDF severity. The present study did not reveal a statistically significant difference in the initial BMI of patients who underwent monomaxillary or bimaxillary surgery. Although the evaluation of DDF severity was not the aim of this study, it is reasonable to assume that patients who underwent monomaxillary surgery had less severe DDF than those who underwent bimaxillary surgery. 

Body weight is the sum of mineral compartments and total body water, glycogen, protein, and fat. However, the body weight of an individual does not reflect the distribution of lean mass, fat, and fluid-it represents only a global measure of all compartments.^15^ Therefore, changes in weight do not reflect which body compartment is affected by malnutrition. However, weight is an important parameter in nutritional assessment because serious unintentional WL is associated with increased morbidity and mortality rates, especially after medium-to-large surgeries. In addition, the rapid loss of large body fat stores strongly indicates a negative energy balance. Previous studies have shown that the success or failure of surgery depends on whether the patient is nutritionally competent;^16^ as such, it is extremely important that surgeons be aware of this aspect to minimize postoperative complications.

One of the factors that may further limit food intake after surgery is intermaxillary fixation for long periods (4-6 weeks).^8^ This problem was routine when fixation with steel wires was used. However, a stable internal fixation eliminates this procedure. In this study, no patient underwent intermaxillary fixation during the postoperative period. Orthodontic elastics were used to guide occlusion for 2-6 weeks; however, patients were able to open their mouth and were instructed how to remove the elastics to feed. 

A previous study found that patients lost an average of 3.07% body fat, and exhibited an average reduction in BMI of 1.63 kg/m^2^ in the four-week postoperative period.^17^ There was no statistically significant difference in WL between male and female patients, nor between those who underwent monomaxillary or bimaxillary surgery.^17^ This is consistent with the present results. Further investigation should be performed to consider other variables, including surgery duration, blood loss, surgical technique (i.e., minimally invasive *versus* conventional), and postoperative complications. 

In this study, it is important to observe that WL at 10 days PO persisted until 40 days PO, which confirms that patients, regardless of group, continued to experience WL, probably due to restrictions on masticatory function and food restrictions recommended by the surgical team until bone repair occurred. At 90 days PO, BMI values were close to preoperative values, indicating that WL had ceased, and there was recovery of body weight to values similar to those observed at preoperative. This is consistent with the normalization of usual food intake. Thus, orthognathic surgery causes only transient changes in weight, returning to baseline levels after two or three months. One of the highlights of the present study is the assessment of patients over a 90-day period, which is unusual in studies that assessed nutritional status and WL without nutritional supplementation.

In the present study, there was no statistically significant difference between the monomaxillary and bimaxillary groups in terms of WL or BMI at any postoperative time point. The authors believe that this is because standard dietary postoperative recommendations were provided to the patients, and the use of intermaxillary elastics was the same for both groups. No complications occurred during the postoperative period. After data evaluation, it can be concluded that the presence of DDF did not necessarily render patients more prone to nutritional deficits. In addition, the surgery induced WL until postoperative day 40. This condition was transient and returned to normal after three months, independent of the type of surgery.

## References

[1] Proffit WR, White RP, Sarver DM (2003). Contemporary treatment of dentofacial deformity..

[2] Palomares NB, Celeste RK, Miguel JA (2016). Impact of orthosurgical treatment phases on oral health-related quality of life. Am J Orthod Dentofacial Orthop.

[3] Bahmanyar S, Namin AW, Weiss 2nd RO, Vincent AG, Read-Fuller AM, Reddy LV (2021). Orthognathic surgery of the mandible. Facial Plast Surg.

[4] Meger MN, Fatturi AL, Gerber JT, Weiss SG, Rocha JS, Scariot R (2021). Impact of orthognathic surgery on quality of life of patients with dentofacial deformity a systematic review and meta-analysis. Br J Oral Maxillofac Surg.

[5] Belusic Gobic M, Kralj M, Harmicar D, Cerovic R, Mady Maricic B, Spalj S (2021). Dentofacial deformity and orthognathic surgery influence on self-esteem and aspects of quality of life. J Craniomaxillofac Surg.

[6] Wolford LM, Chemello PD, Hilliard F (1994). Occlusal plane alteration in orthognathic surgery part I: effects on function and esthetics. Am J Orthod Dentofacial Orthop.

[7] Irgebay Z, Beiriger JC, Beiriger JW, Matinrazm S, Natali M, Yi C (2022). Review of diet protocols following orthognathic surgery and analysis of postoperative weight loss. Cleft Palate Craniofac J.

[8] Ooi K, Inoue N, Matsushita K, Yamaguchi H, Mikoya T, Kawashiri S (2021). Body weight loss after orthognathic surgery comparison between postoperative intermaxillary fixation with metal wire and elastic traction, factors related to body weight loss. J Maxillofac Oral Surg.

[9] Silva AC, O'Ryan F.Poor DB (2006). Postoperative nausea and vomiting (PONV) after orthognathic surgery a retrospective study and literature review. J Oral Maxillofac Surg.

[10] Ooi K, Inoue N, Matsushita K, Yamaguchi HO, Mikoya T, Kawashiri S (2019). Factors related to patients' nutritional state after orthognathic surgery. Oral Maxillofac Surg.

[11] Giacobbo J, Mendel MIL, Borges WD, El-Kik RM, Oliveira RB, Silva DN (2009). Assessment of nutritional anthropometric parameters in adult patients undergoing orthognathic surgery. Rev Odonto Ciênc.

[12] Wolford LM, Rodrigues DB, Limoeiro E (2011). Orthognathic and TMJ surgery postsurgical patient management. J Oral Maxillofac Surg.

[13] Figueiredo LMG, Carvalho MC, Sarmento VA, Brandão GRR, Oliveira TFLd, C B (2013). Evaluation the nutritional status of subjects before and after orthognathic surgery pilot study. Rev Cir Traumatol Buco-Maxilo-Fac.

[14] Sebastiani AM, Baratto-Filho F, Bonotto D, Kluppel LE, Rebellato NL, Costa DJ (2016). Influence of orthognathic surgery for symptoms of temporomandibular dysfunction. Oral Surg Oral Med Oral Pathol Oral Radiol.

[15] Fogarasi A, Gonzalez K, Dalamaga M, Magkos F (2022). The impact of the rate of weight loss on body composition and metabolism. Curr Obes Rep.

[16] Chidyllo SA, Chidyllo R (1989). Nutritional evaluation prior to oral and maxillofacial surgery. N Y State Dent J.

[17] Hammond D, Williams RW, Juj K, O'Connell S.Isherwood G.Hammond N (2015). Weight loss in orthognathic surgery a clinical study. J Orthod.

